# Tau seeds from Alzheimer's disease brains trigger tau spread in macaques while oligomeric‐Aβ mediates pathology maturation

**DOI:** 10.1002/alz.13604

**Published:** 2023-12-26

**Authors:** Morgane Darricau, Changsong Dou, Remi Kinet, Tao Zhu, Li Zhou, Xianglei Li, Aurélie Bedel, Stéphane Claverol, Caroline Tokarski, Taxiarchis Katsinelos, William A. McEwan, Ling Zhang, Ran Gao, Mathieu Bourdenx, Benjamin Dehay, Chuan Qin, Erwan Bezard, Vincent Planche

**Affiliations:** ^1^ Univ. Bordeaux, CNRS Institut des Maladies Neurodégénératives Bordeaux France; ^2^ NHC Key Laboratory of Human Disease Comparative Medicine Beijing Engineering Research Center for Experimental Animal Models of Human Critical Diseases National Center for Technology and Innovation of Animal Model Institute of Laboratory Animal Sciences Chinese Academy of Medical Sciences (CAMS) Beijing P.R. China; ^3^ CHU de Bordeaux Service de biochimie, Bordeaux Univ. Bordeaux Bordeaux France; ^4^ Univ. Bordeaux, Bordeaux Proteome Bordeaux France; ^5^ UK Dementia Research Institute Department of Clinical Neurosciences University of Cambridge Cambridge UK; ^6^ UK Dementia Research Institute UCL Queen Square Institute of Neurology London UK; ^7^ Changping National laboratory (CPNL) Beijing China; ^8^ Motac Neuroscience Floirac France; ^9^ CHU de Bordeaux, Pôle de Neurosciences Cliniques Centre Mémoire de Ressources et de Recherche Bordeaux France

**Keywords:** Alzheimer's disease, beta‐amyloid, non‐human primate, prion‐like, tau, tauopathy

## Abstract

**INTRODUCTION:**

The “prion‐like” features of Alzheimer's disease (AD) tauopathy and its relationship with amyloid‐β (Aβ) have never been experimentally studied in primates phylogenetically close to humans.

**METHODS:**

We injected 17 macaques in the entorhinal cortex with nanograms of seeding‐competent tau aggregates purified from AD brains or control extracts from aged‐matched healthy brains, with or without intracerebroventricular co‐injections of oligomeric‐Aβ.

**RESULTS:**

Pathological tau injection increased cerebrospinal fluid (CSF) p‐tau181 concentration after 18 months. Tau pathology spreads from the entorhinal cortex to the hippocampal trisynaptic loop and the cingulate cortex, resuming the experimental progression of Braak stage I to IV. Many AD‐related molecular networks were impacted by tau seeds injections regardless of Aβ injections in proteomic analyses. However, we found mature neurofibrillary tangles, increased CSF total‐tau concentration, and pre‐ and postsynaptic degeneration only in Aβ co‐injected macaques.

**DISCUSSION:**

Oligomeric‐Aβ mediates the maturation of tau pathology and its neuronal toxicity in macaques but not its initial spreading.

**Highlights:**

- This study supports the “prion‐like” properties of misfolded tau extracted from AD brains.
- This study empirically validates the Braak staging in an anthropomorphic brain.
- This study highlights the role of oligomeric Aβ in driving the maturation and toxicity of tau pathology.
- This work establishes a novel animal model of early sporadic AD that is closer to the human pathology.

## 1 BACKGROUND

Alzheimer's disease (AD) is the most prevalent tauopathy,^1^ characterized by the co‐occurrence of amyloid β (Aβ)‐positive extracellular plaques and intracellular neuronal tau pathology affecting both neurites (neuropil threads and neuritic plaques) and cell‐body (neurofibrillary tangles, NFTs).^2^ The most commonly accepted theoretical pathophysiological framework for AD is based on the amyloid cascade hypothesis and its variants. It postulates a causal link between the aggregation of Aβ, tau pathology, neurodegeneration, and cognitive decline.^3^, ^4^, ^5^ However, this linear deterministic concept, which fits genetic autosomal dominant forms of AD, is challenged in sporadic AD, where tau pathology may precede amyloid pathology.^6^

In vitro,^7^ rodent,^8^, ^9^, ^10^, ^11^ and human tau‐PET studies^12^ have suggested a cell‐to‐cell propagation of AD‐tau pathology. This “prion‐like” hypothesis postulates that misfolded tau assemblies can template the polymerization of nearby soluble tau proteins.^13^ Then, pathological tau aggregates spread in anatomically and functionally connected brain regions and coaggregate with new proteins.^14^, ^15^ The “prion‐like” hypothesis may explain the clinical heterogeneity of AD depending on the initial anatomical pattern of tau deposition and the spatiotemporal trajectories of tau pathology.^16^ Within this conceptual framework, current translational research tries to elucidate whether local replication or distal spread is the rate‐determining step of tau seed accumulation and symptoms progression in AD and what factors influence this progression.^17^, ^18^

The nature of the synergistic interaction between Aβ and tau is a matter of debate in AD. Still, neuropathological studies in humans support that tau pathology extends beyond the initially affected brain regions only when amyloid pathology is also present.^19^ Recent findings in transgenic mice injected with patient‐derived tau seeds or AD brain homogenates suggest that Aβ plaques may be “niches” facilitating the amplification of proteopathic tau seeds and their initial aggregation in neuritic plaques.^20^, ^21^ Other findings suggest that soluble oligomeric Aβ (but not plaques) would be the Aβ species interacting with tau at the synaptic level, initiating tau hyperphosphorylation and misfolding.^22^, ^23^ This toxic role for protofibrils/oligomers has been recently underlined by the positive results of the CLARITY‐AD phase 3 clinical trial, showing that lecanemab (a monoclonal antibody selectively targeting large soluble Aβ protofibrils) reduces both cognitive decline and the regional progression of tau‐PET in patients with early AD.^24^ On the other hand, immunotherapies targeting specifically Aβ plaques, such as donanemab, exhibit comparable therapeutic properties while not impacting tau progression measured with PET.^25^ Thus, a better understanding of the Aβ‐tau toxic relationship is necessary to design future anti‐amyloid therapies targeting the most pathogenic amyloid species.

The study of AD pathophysiology in rodents has led to some disappointments, particularly in developing new therapies, leading to the posit that the testbed species matter. The rhesus monkey (*Macaca mulatta*) is the phylogenetically closest species to humans on which we can conduct research. Unlike rodents, macaques share more than 98% homology with humans for tau protein and almost 100% homology for Aβ peptide sequences.^26^ Macaques and humans expressed 3R and 4R tau isoforms, both aggregated in AD, while adult wild‐type rodents only expressed 4R tau. Another significant divergence concerns the amino‐terminal region of tau, where rodents lack a primate‐specific motif spanning residues 18–28, which mediates tau interaction with many neuronal proteins.^27^ Moreover, investigating tau's complex cell‐to‐cell anatomical propagation is limited in rodents’ brains, while macaques offer anthropomorphic cerebral shape and connectivity.^28^ Thus, the experimental study of tau seeding and spreading, and its interaction with Aβ, is urgently required in macaques to validate or refute theoretical models in sporadic AD, such as the Braak staging, the amyloid cascade hypothesis, or the prion‐like properties of misfolded tau.

To address these critical issues, we first purified and characterized sarkosyl‐insoluble tau proteopathic seeds extracted from the brains of AD patients (AD‐tau) or extracts obtained from the brains of healthy aged‐matched individuals (CTL‐tau). We then injected 17 rhesus macaques with AD‐tau, CTL‐tau, or sham injections into the entorhinal cortex, where AD tauopathy is supposed to start in humans (Braak stage I‐II).^29^ Experimental sub‐groups of macaques also received recombinant oligomeric Aβ peptide injections in lateral ventricles or sham injections. After cerebrospinal fluid (CSF) collections, macaques were terminated 18 months after tau injections to perform neuropathological studies and proteomic analysis.

## 2 METHODS

### 2.1 Tau extraction from human brains

Human brain samples were obtained from the Netherlands Brain Bank (Department of Pathology, Amsterdam UMC) and the French Brain Bank GIE NeuroCEB (Pitié‐Salpétrière Hospital, Paris). Twelve amygdala, hippocampal, or neocortical (superior frontal, cingulate, or parahippocampal gyrus) samples were dissected from fresh‐frozen *post mortem* tissue from patients (82–97 years old) with histologically confirmed sporadic AD, with Braak stages V or VI on neuropathological examination. We also selected six samples from age‐matched non‐demented brain donors (86–91 years old) with no or low AD neuropathological changes (Braak stages ≤II) (Table S1).

**RESEARCH IN CONTEXT**

1. **Systematic review**: The authors reviewed the literature using traditional sources. In vitro, rodent, and human tau‐PET studies had previously suggested a cell‐to‐cell propagation of tau pathology in AD. Furthermore, transgenic and seeding animal models have attempted to elucidate the nature of the synergistic interaction between Aβ and tau. However, conclusions remain debated. The administration of human brain‐derived tau aggregates to macaques (the phylogenetically closest species to humans on which we can conduct research) is advocated as a tool for understanding the pathological development of the diseases from which they originate and the subsequent pathophysiology. Such studies may help address the methodological limitations of previous experimental work.
2. **Interpretation**: The injection of nanograms of tau proteopathic seeds purified from AD brains into the entorhinal cortex of macaques experimentally supports Braak's tauopathy progression model. It grounds the “prion‐like” hypothesis of AD tauopathy in an anthropomorphic brain, and establishes a new animal model closely resembling early sporadic AD in humans. Histological and proteomic analyses suggest that simultaneous injections of oligomeric Aβ may contribute to the maturation of tau pathology rather than in its ability to replicate and spread.
3. **Future directions**: Although typical of AD tauopathy, the lesions induced by our protocol remain sparse, and this primate model could be optimized. The behavioral impact of tau seeds injections in macaques (with or without concomitant oligomeric Aβ injections) should also be studied in future experiments.

**FIGURE 1:**
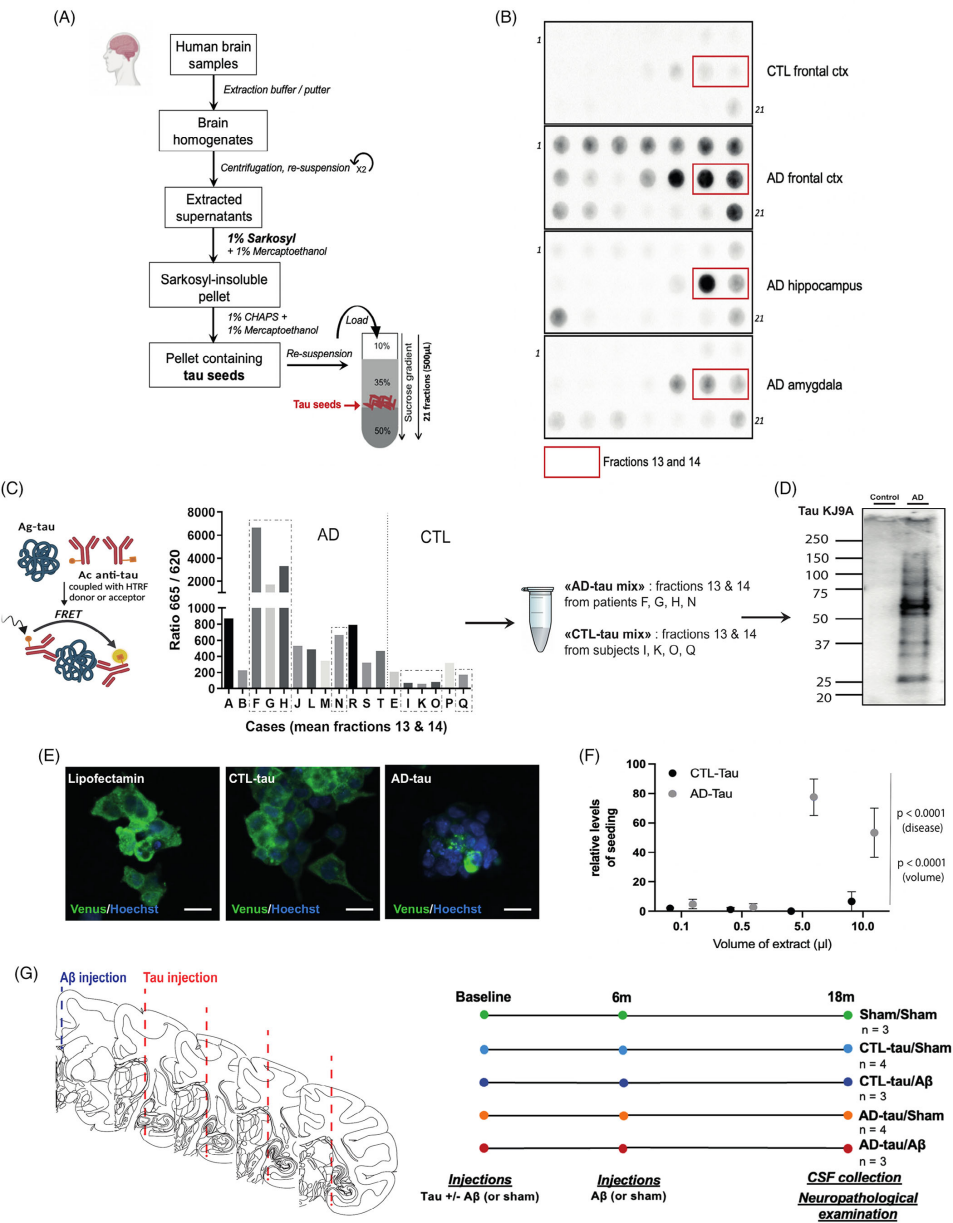
Tau seeds extraction, selection, and characterization. (A) Schematic summary of the extraction/purification protocol used to purify tau aggregates from *post mortem* Alzheimer's disease (AD) and control (CTL) brains. The final sucrose gradient purification procedure leads to the isolation of 21 fractions of 500 μL. (B) Examples of filter retardation assay probed with AT8 antibody to assess the presence of pathological phospho‐tau aggregates in the different fractions isolated in the sucrose gradient. Red rectangles indicate the fractions selected to prepare AD and CTL samples (fractions 13 and 14, where tau aggregates are most frequently found, according to Greenberg et al.^30^). (C) Schematic representation of the HTRF® Tau aggregation assay (left panel). Fluorescence signal intensity reflects the number of tau aggregates in each 12 AD or 6 CTL samples (pooled fractions 13 and 14) screened for this study (middle panel). Based on this assay, we pooled the samples from four AD patients (F, G, H, and N) and four CTL (I, K, O, and Q) to obtain an “AD mix” and a “CTL mix” (right panel) that were further characterized and used for primates experiments. Dotted rectangles indicate the selected samples. (D) Western blot characterization of “AD‐tau mix” and “CTL‐tau mix” solutions using the pan‐tau KJ9A antibody. (E) Representative fluorescent images of the high throughput tau seeding assay using the tau P301S‐Venus cell line transfected with AD‐mix or CTL‐mix. Scale bar: 50 μm. (F) Quantitative results of the assay (number of tau‐venus‐positive punctae/aggregates per cell, 48 h after adding seeds or control material). The *p*‐values refer to the results of a two‐way analysis of variance (ANOVA). (G) Schematic and timeline of the macaque experiments with the presentation of the five experimental conditions

Sarkosyl‐insoluble tau aggregates were extracted and purified according to an adaptation of the Greenberg et al. protocol.^30^ Briefly, each brain sample (∼1 g of tissue) was homogenized in 10 mL of ice‐cold extraction buffer (10 mM Tris‐HCl, 0.8 M NaCl, 10% sucrose, 1 mM EGTA, and complete™ Mini Protease Inhibitor Cocktail (Merck)) using a glass/Teflon homogenizer. Homogenates were then centrifuged at 27,200 *g* for 20 min at 4°C. Pellets were re‐extracted using the same conditions, and the supernatants from all two extractions were combined. Additional sarkosyl and 2‐mercaptoethanol were added to the pooled supernatant to reach a 1% final concentration. After 2 h of incubation (200 rpm shaking) at 37°C, samples were ultracentrifuged at 26,800 rpm for 35 min at room temperature (Beckman SW41Ti rotor). Supernatants were discarded, and the insoluble pellets containing pathological tau aggregates were resuspended in 5 mL extraction buffer containing 1% 2‐mercaptoethanol and 1% 3‐[(3‐cholamidopropyl)dimethylammonio]−1‐propanesulfonate (CHAPS) and ultracentrifuged again (26,800 rpm for 1 h at room temperature in SW41Ti rotor). Supernatants were discarded. Tau‐containing pellets were resuspended in 3 mL extraction buffer containing 0.1% 2‐mercaptoethanol and layered over a sucrose step gradient consisting of 4 mL of 50% sucrose and 3.5 mL of 35% sucrose in extraction buffer with 0.1% 2‐mercaptoethanol. After centrifugation for 2 h at 26,800 rpm in an SW41Ti rotor, 21 fractions of 500 μL were collected from the sucrose gradient (Figure 1A) from the top (fraction 1) to the bottom (fraction 21) and stored at −80°C until used for further analysis.

### 2.2 Characterization and selection of AD‐tau seeds

For each sample, the 21 fractions were analyzed for the presence of pathological phospho‐tau aggregates by filter retardation assay on nitrocellulose membrane using AT8 antibody (Fischer Scientific, MN1020), as previously described^31^ (Figure 1B). Based on this assay and previous findings,^30^ fractions 13 and 14 were selected for further characterization using the HTRF tau aggregation kit (Cisbio, #6FTAUPEG) according to the manufacturer's conditions. This assay uses specific monoclonal antibodies and fluorescence resonance energy transfer (FRET) to detect tau aggregation induced by biological samples. Based on the results of this assay (Figure 1C), we selected and pooled four AD samples with the highest FRET signal intensity (“AD‐tau mix”) and four control samples with the lowest FRET signal intensity (“CTL‐tau mix”) to have a sufficient amount of materials, ready for in vivo injections.

The “AD‐tau mix” and the “CTL‐tau mix” were further analyzed with Western‐Blot using pan‐tau KJ9 antibody (Agilent) (Figure 1D). Tau concentrations in the “AD‐tau mix” and “CTL‐tau mix” were measured with quantitative filter retardation assay on nitrocellulose membranes and HT7 antibody (Fischer Scientific). The standard curve for quantification was obtained using serial two‐fold dilutions of a recombinant tau solution at 1.5 mg/mL (human 6xHis‐0N4R tau P301S, initially expressed in *E. coli* BL21 DE3, New England Biolabs, as described previously).^32^ The total tau concentration in the “AD‐tau mix” was 115 ng/mL.

Before in vivo experiments, the in vitro seeding ability of the “AD‐tau mix” and the “CTL‐tau mix” was assessed with a high‐throughput seeding assay using clonal 0N4R tau P301S‐Venus HEK293 cells, as previously described.^33^ Briefly, tau extracts were diluted in OptiMEM (Life Technologies), mixed with Lipofectamine 2000, left for 10 min at room temperature (RT), and then administered to the cells. After 1 h, c‐DMEM was added to each well to stop the seed transduction. The cells were incubated at 37°C for 48 h after adding tau extracts and then fixed with ice‐cold methanol for 3 min at RT. Nuclei were stained with Hoechst, and images were acquired at 405 and 488 nm with an InCell Analyser 6000 high‐resolution automated microscope. Nuclear and seeded aggregates counting was performed using the Fiji software.^34^ The relative level of seeding was calculated as the number of Venus‐positive punctate/aggregates in each field, normalized to the corresponding number of cells, and compared to the untreated control. (Figure 1E,F)

### 2.3 Macaques and stereotactic injections

Seventeen female and male rhesus macaques (*Macaca mulatta*, Xierxin, Beijing, China) were used in this study. In order to obtain unbiased results regarding the age, sex, or weight of the animals, they were assigned to an experimental group after randomization (Table 1 and Table S2 for individual details). They were fed with fruit, vegetables, and monkey pellets. Water was available ad libitum. Animal care was supervised daily by veterinarians skilled in the healthcare and maintenance of non‐human primates. Experiments were performed following the European Union directive (2010/63/EU) on protecting animal use for scientific purposes in the Institute of Laboratory Animal Science in Beijing, accredited by AAALAC (Association for Assessment and Accreditation of Laboratory Animal Care). Experimental procedures were performed following study design acceptance by two independent ethics committees in France (CEEA 50, #12286) and China (Institute of Lab Animal Science, Chinese Academy of Science, Beijing, approval number: QC18008). Following the recommendations of the Weatherall report, macaques were individually housed, but with the possibility to interact with adjacent macaques under controlled environmental conditions of humidity (50 ± 5%), temperature (24 ± 1°C), and light (12 h light/12 h dark cycles).

**TABLE 1:** Population characteristics.

	Sham/sham (*n* = 3)	CTL‐tau/Sham (*n* = 4)	CTL‐tau/Aß (*n* = 3)	AD‐tau/sham (*n* = 4)	AD‐tau/Aß (*n* = 3)	*p*‐Value
Sex (M/F)	2/1	2/2	1/2	2/2	1/2	0.94^a^
Weight, kg; mean (SD)	6.1 (1.3)	6.9 (1.5)	6.3 (1.8)	6.5 (1.3)	6.2 (1.1)	0.92^b^
Mean age, years; mean (SD)	14.7 (1.5)	15.0 (0.8)	14.7 (1.2)	15.0 (1.4)	15.2 (0.6)	0.88^b^

*Note*: The animals were randomly distributed into the five groups. As expected, there were no differences between the groups in terms of sex, weight, or age.

^a^ Chi‐squared test.

^b^ Kruskal–Wallis test.

Macaques were randomly selected to receive 200 μL of “AD‐tau mix,” “CTL‐tau mix,” or sham solution (15% sucrose‐solution). Solutions were bath‐sonicated for 5 min before inoculations. Bilateral injections were performed at four rostrocaudal levels of the entorhinal cortex (25 μL per injection site) (Track 1: anterior commissure (AC) = −8, length (L) = 9, depth (D) = −7; Track 2: AC = −10, L = 8.5, D = −6; Track 3: AC = −12, L = 8.5, D = −5, and Track 4: AC = −13, L = 7, D = −3). After each injection, the syringe was left in place for 5 min to avoid leakage along the needle track. Subgroups of macaques also received intracerebroventricular (ICV) injections of oligomeric Aβ. Recombinant Aβ was bought from Eurogentec (#AS‐20276, Bachem). Dehydrated oligomers were prepared as previously described,^35^ resuspended in NH4‐acetate (100 μg in 100 μL, 50 mM, pH 7.3), and incubated at 37°C for 30 min (shaking) before injection. Animals received 100 μL of oligomeric Aβ solution at baseline and after 6 months. Finally, three macaques received both “AD‐tau mix” and oligomeric Aβ (AD‐tau/Aβ), four macaques “AD‐tau mix” only (AD‐tau/sham), three macaques “CTL‐tau mix” and oligomeric Aβ (CTL‐tau/Aβ), four macaques “CTL‐tau mix” only (CTL‐tau/sham) and three macaques received sham injections (sham/sham) (Figure 1G).

Eighteen months post‐injections, monkeys were euthanized by sodium pentobarbital overdose (150 mg/kg iv). This was followed by perfusion with 2 L of 0.9% saline solution and 1% heparin, as the European Veterinary Medical Association guidelines recommended. Brains were quickly removed after perfusion and then dissected along the midline. The left hemisphere was immediately freshly frozen by immersion in a cold isopentane bath at −50°C for at least 5 min and stored at −80°C for biochemical investigation. The right hemisphere was immediately post‐fixed in 4% PFA for a week, cryo‐protected in sucrose PBS until sunk, frozen in a snap‐frost isopentane bath at −50°C, and stored immediately at −80°C until sectioning for histological analyses.

### 2.4 CSF collection and analysis

Eighteen months post‐injections, lumbar punctures were performed immediately before brain perfusion to collect 1.5 mL of CSF in polypropylene tubes, frozen, and stored at −80°C. CSF samples were analyzed at Bordeaux University Hospital using clinical‐grade procedures on a Lumipulse G600II instrument (Furjirebio). The lumipulse G β‐amyloid 1‐42, G β‐amyloid 1‐40, G total‐tau, and G phospho‐tau (p‐tau181) commercial kits were used per the manufacturer's protocol.

### 2.5 Histological analysis: tau, Aβ pathology, and neuroinflammation

Immunohistochemical staining for phosphorylated tau (AT8, Fischer Scientific, 1:500, mouse), Aβ (6E10, BioLegend, 1:500, mouse), microglia (Iba1, ab5076, Abcam, 1:1000, goat), and astrocytes (mix of GFAP and S‐100, MAB 3402 GA5, Sigma, 1:2000 and PAP 11341, Abcam, 1:1000, mouse) were performed on free‐floating coronal slices (40 μm thickness), as previously described.^36^ Briefly, sections were incubated with primary antibodies overnight at RT and then with an appropriate anti‐species peroxidase EnVision‐HRP system (anti‐mouse Agilent DAKO or anti‐goat Vector Laboratories) for 30 min. Sections were revealed with 3,3′diaminobenzidine (DAB, Agilent DAKO), counterstaining with 0.1% Cresyl violet solution, and then mounted on gelatinized slides. For quantitative analyses, all sections were scanned in a panoramic digital slide scanner (Panoramic Scan II, 3DHISTECH).

For the analysis of tau and Aβ pathology, quantification was performed on two series of two slices per animal at ×20 magnification in the pre‐specified region of interest: the injection site (entorhinal cortex), connected brain areas (dentate gyrus, CA1, subiculum, and cingulate cortex) and non‐connected brain regions (somatosensory cortex and putamen). AT8‐positive tangles, neuropil threads, and Aβ plaques were counted manually and normalized by the surface of the delineated area of interest. Microglia reactivity was assessed using microglial morphology analyses through fractal dimension quantification based on microscopic acquisitions of Iba1 staining (15 images/animal/region of interest), as previously described.^37^ Astrocytic activation was estimated by GFAP/S100, the quantification of the immunostaining‐positive surface with the Mercator software (ExploraNova, France). The evaluator was blinded to the experimental conditions for all quantitative analyses.

To observe Aβ plaques and mature neurofibrillary tangles, thioflavin‐S staining was performed on coronal free‐floating slices (40 μm thickness). Tissues were washed three times with PBS‐1X and stained with 0.05% thioflavin‐S (Sigma, T1892‐25G) diluted in distilled water for 6 min. They were washed three times with 80% ethanol for 10 min and three times with PBS‐1X for 10 min. Then, sections were counterstained with 10 μM of Hoechst 33258 and mounted with Vecta‐Shield without Dapi media (Vector Laboratories) on non‐gelatinized slides. Images were acquired using Zeiss Axio Imager 2 (ExploraNova) at ×40 magnification.

### 2.6 Histological analysis: Neurodegeneration

Serial sections of the hippocampus corresponding were incubated with an anti‐NeuN antibody (MAB 377, Merck Millipore, 1:1000, mouse) overnight at RT and then with EnVision‐HRP enzyme conjugate secondary antibody (anti‐mouse, Agilent DAKO) for 30 min. Sections were revealed with 3,3′ diaminobenzidine (DAB, Agilent DAKO), counterstaining with 0.01% Nissl solution, and mounted on gelatinized slides. NeuN‐positive cells were counted by stereology using Leica DM6000B motorized microscope coupled with the Mercator software (ExploraNova, France), as previously described.^38^ Quantification was performed in the stratum pyramidal and the stratum radiatum lacunosum molecular (SRLM) of CA1. Areas were delineated for each slide, and probes for stereological counting were applied to the obtained map (size of probes: 100 × 80 μm spaced by 400 × 300 μm). Finally, the optical fractionator method was used to estimate each macaque's total number of NeuN‐positive cells. The evaluator was blinded to the experimental conditions.

### 2.7 Immunoblotting: Synaptic proteins

Tissue patches from CA1 were collected on 300‐μm thick cryostat‐cut sections (*n*  =  5 patches per animal). Patches were pooled and mechanically lysed on ice with 100 μL of RIPA buffer (50 mM tris‐HCl, 150 mM NaCl, 1.0% Triton X‐100, 0.5% Na‐deoxycholate, and 0.1% SDS) with a protease and phosphatase inhibitor cocktail (Complete Mini, Roche Diagnostics). Lysates were sonicated in a water bath for 10 min, then incubated on ice for 20 min and centrifuged at 15,000 *g* for 15 min at 4°C. Supernatants were collected, and the total amount of protein was determined by bicinchoninic acid (BCA) assay. Based on total protein concentrations calculated from BCA assays, aliquots of tissue lysates corresponding to known amounts of total protein per sample were prepared for each animal in Laemmli buffer (Tris‐HCl 25 mM pH 6.8, glycerol 7.5%, SDS 1%, DTT 250 mM, and bromophenol blue 0.05%) for immunoblotting experiments.

Western blots were run using 20 μg of protein separated by sodium dodecyl sulfate‐polyacrylamide gel electrophoresis (SDS‐PAGE) (8%) and transferred to nitrocellulose membranes. Membrane incubation with sequential primary antibodies was performed 1 h at RT with first mouse anti‐synaptophysin (1:500, Abcam Sy38, ab8049), second mouse anti‐PSD95 (1:1000, Merck Millipore, MABN68) and finally mouse anti‐β‐actin (1:10 000, Sigma, A5441) to control equal loading in the third step. A Super Signal West Chemiluminescent kit revealed appropriate secondary antibodies coupled to peroxidase (Immobilon Western, Chemiluminescent HRP substrate, Millipore). Chemiluminescence images were acquired using the ChemiDoc + XRS system measurement (Bio‐Rad). Signals per lane were quantified using ImageJ software. A ratio (protein of interest normalized to β‐actin protein expression) of the signal on loading animal was calculated and used in statistical analyses. Each immunoblot was performed in duplicate in two separate experiments, and the average value for each animal was calculated for subsequent statistical analyses.

### 2.8 Proteomic analyses: Sample preparation and protein digestion

Tissue patches from the entorhinal cortex and CA1 were collected on 300‐μm thick cryostat‐cut sections (*n*  =  15 patches per structure and animal). Patches were pooled and mechanically lysed on ice with 100 μL of RIPA buffer with a protease and phosphatase inhibitor cocktail. Lysates were incubated for 30 min and then centrifuged at 15, 000 *g* for 15 min at 4°C. Supernatants were collected, and the total amount of protein in the lysates was assessed by bicinchoninic acid assay (BCA) before storage at −80°C. Protein samples were solubilized in Laemmli buffer, and 5 μg per sample were deposited onto SDS‐PAGE gel (10% acrylamide) for separation, concentration, and cleaning purposes. After colloidal blue staining, bands were cut from the gel into 1 mm × 1 mm gel pieces. Gel pieces were unstained in 25 mM ammonium bicarbonate 50% acetonitrile (ACN), rinsed twice in ultrapure water, and shrunk in ACN for 10 min. After ACN removal, gel pieces were dried at room temperature, covered with the trypsin solution (10 ng/μL in 50 mM NH_4_HCO_3_), rehydrated at 4°C for 10 min, and incubated overnight at 37°C. Samples were then incubated for 15 min in 50 mM NH_4_HCO_3_ at room temperature with rotary shaking. The supernatant was collected, and an H_2_O/ACN/HCOOH (47.5:47.5:5) extraction solution was added to gel slices for 15 min. The extraction step was repeated twice. Supernatants were pooled and dried in a vacuum centrifuge. Digests were finally solubilized in 0.1% HCOOH.

### 2.9 Proteomic analyses: nanoLC‐MS/MS analysis and label‐free data analyses

The peptide mixture was analyzed on an Ultimate 3000 nanoLC system (Dionex, Amsterdam, The Netherlands) coupled to an Electrospray Orbitrap Fusion™ Lumos™ Tribrid™ Mass Spectrometer (Thermo Fisher Scientific, San Jose, CA). Ten microliters of peptide digests were loaded onto a 300 μm inner diameter × 5 mm C_18_ PepMap™ trap column (LC Packings) at a flow rate of 10 μL/min. The peptides were eluted from the trap column onto an analytical 75 mm id × 50 cm C18 Pep‐Map column (LC Packings) with a 4%−25% linear gradient of solvent B in 71 min (solvent A was 0.1% formic acid and solvent B was 0.1% formic acid in 80% ACN) followed by a 14 min gradient from 25% to 90% solvent B. The separation flow rate was set at 300 nL/min. The mass spectrometer operated in positive ion mode at a 1.9 kV needle voltage. Data were acquired using Xcalibur 4.1 software in a data‐dependent mode. MS scans (*m/z* 375−1500) were recorded at a resolution of *R*  =  120,000 (@ m/z 200), and an automated gain control target of 4 × 10^5^ ions was collected within 50 ms. Dynamic exclusion was set to 30 s, and top speed fragmentation in Higher‐energy collisional dissociation (HCD) mode was performed over a 3 s cycle. MS/MS scans with a target value of 5 × 10^4^ ions were collected in orbitrap with a maximum injection time of 54 ms and a resolution of 30,000 (@ m/z 200). Additionally, only +2 to +7 charged ions were selected for fragmentation. Other settings were as follows: no sheath nor auxiliary gas flow; heated capillary temperature, 275°C; normalized HCD collision energy of 28% and an isolation width of 1.6 m/z. Monoisotopic precursor selection (MIPS) was set to peptide, and an intensity threshold was set to 2.5 × 10^4^.

Data were searched by SEQUEST through Proteome Discoverer 2.5 (Thermo Fisher Scientific Inc.) against the *Macaca mulatta* protein database (v2021‐03; 44,389 entries). Spectra from peptides higher than 5000 Da or lower than 350 Da were rejected. The search parameters were as follows: mass accuracy of the monoisotopic peptide precursor and peptide fragments was set to 10 ppm and 0.02 Da, respectively. Only b‐ and y‐ions were considered for mass calculation. Oxidation of methionines (+16 Da), methionine loss (‐131 Da), methionine loss with acetylation (‐89 Da), and protein N‐terminal acetylation (+42 Da) were considered as variable modifications.

In comparison, carbamidomethylation of cysteines (+57 Da) was considered a fixed modification. Two missed trypsin cleavages were allowed. Peptide validation was performed using the Percolator algorithm, and only “high confidence” peptides were retained, corresponding to a 1% False Positive Rate at the peptide level. Peaks were detected and integrated using the Minora algorithm embedded in Proteome Discoverer. Proteins were quantified based on unique peptide intensities. Normalization was performed based on the total protein amount. Protein ratios were calculated as the median of all possible pairwise peptide ratios.

### 2.10 Proteomic analyses: Results processing

Proteomic data were analyzed using Python (Python software foundation v.3.9.7 available at https://www.python.org/) and the scientific python stack: scipy (v.1.9.3), numpy (v.1.23.4), matplotlib (v. 3.6.2), and seaborn (v. 0.12.1). Only proteins listed as “Master Protein” (i.e., reviewed proteins in the Uniprot database), with more than one unique peptide and not belonging to a contaminant protein list (including keratin, trypsin, and intermediate filament rod domain‐containing protein types) were kept for the data analysis. Differentially expressed proteins were selected according to (i) a *p*‐value higher than ‐log10(0.01) and (ii) a fold change greater or equal to 0.5. Principal component analysis (PCA) was performed using scikit‐learn module following *Z* score normalization of intensity values. The correlation map between PCA values and neuropathological assessments was done by performing a Pearson correlation between the individual scores in each principal component with the individual values of histological analysis. To ensure a consistent interpretation of the results, an outlier CTL‐Tau animal was removed from entorhinal proteomic analyses due to its abnormal profile (fully segregated from the other samples in the PCA analysis). The principal component further analyzed in the network‐type analysis was selected regarding the positive or negative correlation with histological analysis, the percentage of explained variance, the discrimination profile of principal component scores between control and experimental groups, and the results obtained in network analysis. Network analysis of PCA results was performed using Cytoscape (v.3.9.1),^39^ ClueGO (v.2.5.9),^40^ and CluePedia (v.1.5.9)^41^ with the 10% proteins (under 5th and above 95th percentiles) contributing the most to the selected principal component.

### 2.11 Statistical analysis

Regarding the biochemical characterization of our samples, the effect of different volumes of AD‐tau mix and CTL‐tau mix on the seeding assay was analyzed using a two‐way ANOVA (Figure 1).

After randomizing the monkeys into the different experimental groups, we ensured the absence of differences in terms of age, sex, or weight between the groups using a chi‐squared test or a Kruskal−Wallis test, as appropriate (Table 1).

For descriptive purposes, the results of the analyses conducted in the CSF, the quantitative results from histology and immunoblot experiments were first presented with histograms and individual plots for each experimental group (and for each brain region studied, if applicable). In this study, we chose to have many experimental groups (*n* = 5) to control our experiments better. The trade‐off was fewer animals per group (*n* = 3 or 4). Since our a priori objective was to study the impact of AD‐tau injection and its potential interaction with Aβ, we pooled the different control groups (Sham/sham, CTL‐tau/sham, and CTL‐tau/Aβ) to compare them to the AD‐tau and AD‐tau/Aβ groups (except for the quantification of Aβ plaques where we compared Aβ‐injected animals to all non‐injected animals). In this regard, data were also analyzed and represented with graphics that emphasized the effect size, allowing statistical analyses beyond p‐value,^42^ by using estimation graphics called “Gardner‐Altman plots” as previously reported.^43^ This plot used two graphs. The graph on the left compared one pathological condition to the controls as scatter plots showing the observed individual values along with the descriptive statistics. The graph on the right displays the effect size by presenting the difference distribution between groups using resampled distributions of observed data (color curves). Horizontally aligned with the mean of the test group, the mean difference is indicated with the black circle. The black vertical line illustrates the 95% CI of the mean difference. We further used one‐tailed Mann‐Whitney tests to analyze the two experimental groups in these plots, to provide information about p‐values. We also tested the correlations of histological or biochemical variables using Spearman tests.

All values were expressed as the mean ± standard error of the mean (SEM). Statistical analyses were performed with GraphPad Prism 10.0.3 (GraphPad Software, Inc., San Diego, CA).

### 2.12 Data and material availability

Raw data supporting the results reported in this article are available from the corresponding authors upon reasonable request. The mass spectrometry proteomics data have been deposited to the ProteomeXchange Consortium via the PRIDE partner repository with the dataset identifier PXD040522. The Python code required to process proteomic data has been deposited on GitHub.

## 3 RESULTS

### 3.1 Extraction, purification, and characterization of tau seeds from human AD brains

Sarkosyl‐insoluble tau proteopathic seeds were extracted from 12 amygdala, hippocampal, or cortical human AD brain samples and then purified and selected on a sucrose gradient (AD‐tau) (Figure 1A). The same fractions were extracted from six amygdala or cortical samples from non‐demented age‐matched controls (CTL‐tau). After fractionation on a discontinuous sucrose gradient, pathologically phosphorylated tau was consistently detected in fractions 13 and 14 from AD brains using AT8 filter retardation assay (Figure 1B), as previously described with this extraction protocol.^30^ Thus, fractions 13 and 14 for each AD case or CTL sample were pooled for further characterization with an aggregation assay, and tau concentrations were assessed with a quantitative HT7 filter retardation assay. Based on these two assays, we selected samples from four AD patients with the highest concentrations of tau aggregates and from four CTL samples to constitute a pooled AD‐tau mix and a CTL‐tau mix (Figure 1C). The final total‐tau concentration was higher in the AD‐tau mix (115 ng/mL) than in the CTL‐tau mix (<10 ng/mL). A Western blot with the KJ9A pan‐tau antibody confirmed that the AD‐tau mix was enriched in many tau species and isoforms compared to the CTL‐tau mix (Figure 1D). Finally, the incubation of AD‐tau and CTL‐tau on P301S tau‐venus expressing cells^33^ revealed substantially higher in vitro seeding activity of the AD‐tau mix compared to the CTL‐tau mix, with a dose‐response effect (Figure 1E,F; two‐way ANOVA, disease (AD‐tau vs. CTL‐tau): *F* = 32.7, *p* < 0.0001; volume: *F* = 11.0, *p* < 0.0001), confirming an enrichment of seeding competent species in the AD‐tau mix.

### 3.2 Macaque cohort

Seventeen female and male mature (13−17 years old) rhesus macaques were randomly injected in the entorhinal cortex with AD‐tau, CTL‐tau, or sham injections. Experimental sub‐groups of macaques also received two injections (6 months apart) of recombinant oligomeric Aβ peptides in lateral ventricles (AD‐tau/Aβ and CTL‐tau/Aβ groups), or sham injections (AD‐tau/sham and CTL‐tau/sham groups) (Figure 1G). As expected after randomization, the experimental groups did not differ in terms of age, sex, or weight at baseline (*p* > 0.88, Table 1, and Table S2 for individual information).

The oligomeric Aβ preparation used in this study has been extensively characterized in vitro and validated in vivo in previous work from our group and others.^35^, ^44^, ^45^ In these experimental conditions, oligomeric Aβ can diffuse in several cortical and sub‐cortical regions in macaques,^46^, ^47^ allowing its interaction with tau.

### 3.3 CSF biomarkers of tauopathy and neurodegeneration

**FIGURE 2:**
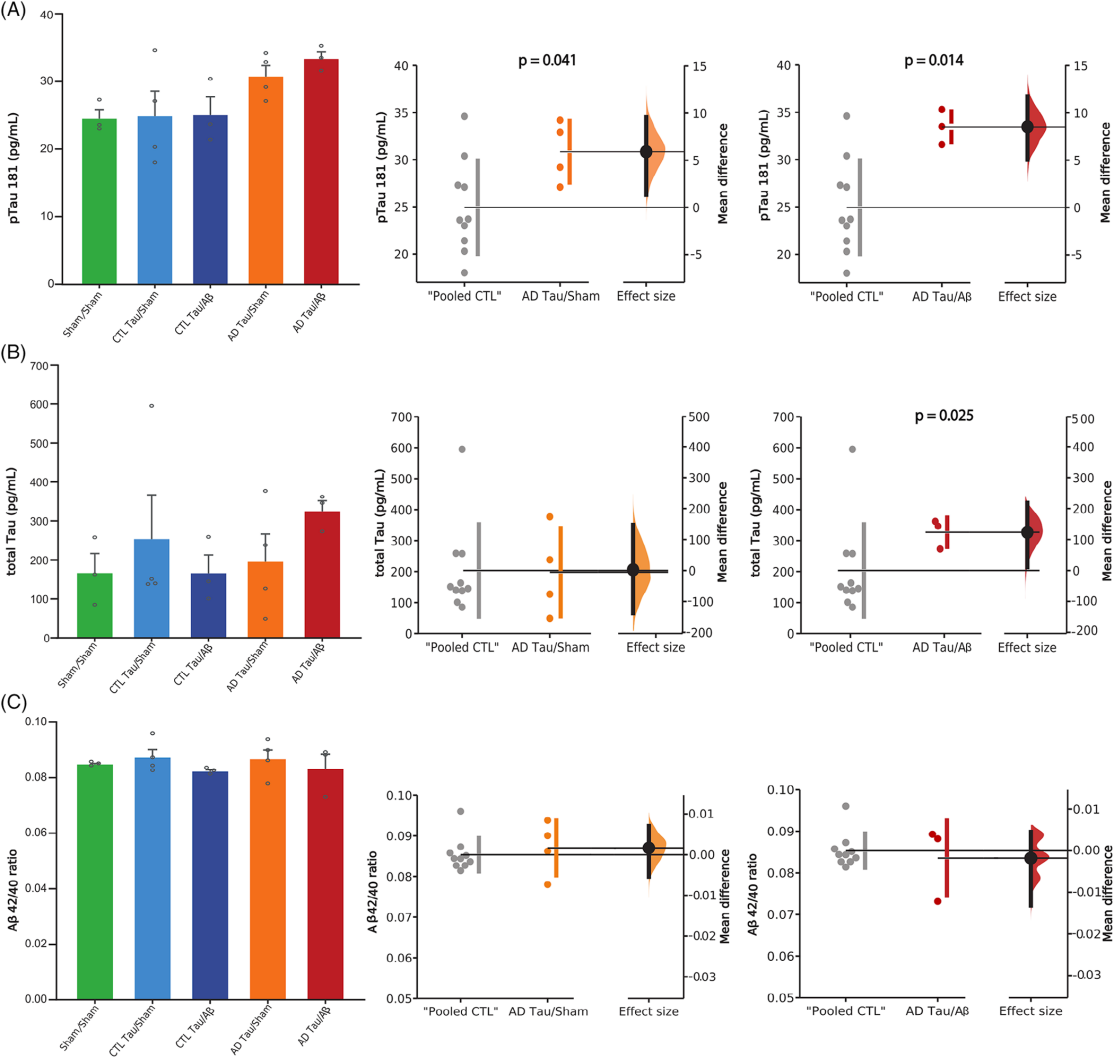
Injections of Alzheimer's disease (AD) patients‐derived tau aggregates increased cerebrospinal fluid (CSF) ptau‐181 and total‐tau concentrations in macaques. Quantification of p‐tau181 (A), total tau (B), and amyloid‐β (Aβ) 42/40 ratio (C) concentrations 18 months after injection with the clinical‐grade Lumipulse technology (Fujirebio). Quantitative results are represented in the five experimental groups using bar and dot plots. They are also represented with estimation plots where sham animals and the two control (CTL)‐tau groups were pooled (“pooled CTL”) and compared to AD‐tau/sham or AD‐tau/Aβ groups. The *p*‐values refer to the results of Mann‐Whitney tests

Because soluble tau can be easily detected in body fluids,^48^ we first measured total‐tau and p‐tau181 concentrations in CSF. The CSF was collected 18 months post‐injection by lumbar puncture in clinical‐grade conditions, and AD biomarkers were measured with the Lumipulse immunoassays, as in clinical routine. We found a significant increase in the concentration of p‐tau181 (a marker of tauopathy) in the CSF of both AD‐tau/sham and AD‐tau/Aβ macaques, with a more robust effect size in the animals co‐injected with oligomeric Aβ (Figure 2A; Mann‐Whitney test: pooled‐CTL vs. AD‐tau/sham, *p* = 0.041; pooled‐CTL vs. AD‐tau/Aβ, *p* = 0.014). We also found higher total‐tau concentrations (a marker of neurodegeneration) in AD‐tau/Aβ macaques (Figure 2B; Mann‐Whitney test: “pooled‐CTL” vs. AD‐tau/Aβ, *p* = 0.025). There was no difference between groups for the Aβ42/40 ratio (Figure 2C).

### 3.4 Tau pathology

**FIGURE 3:**
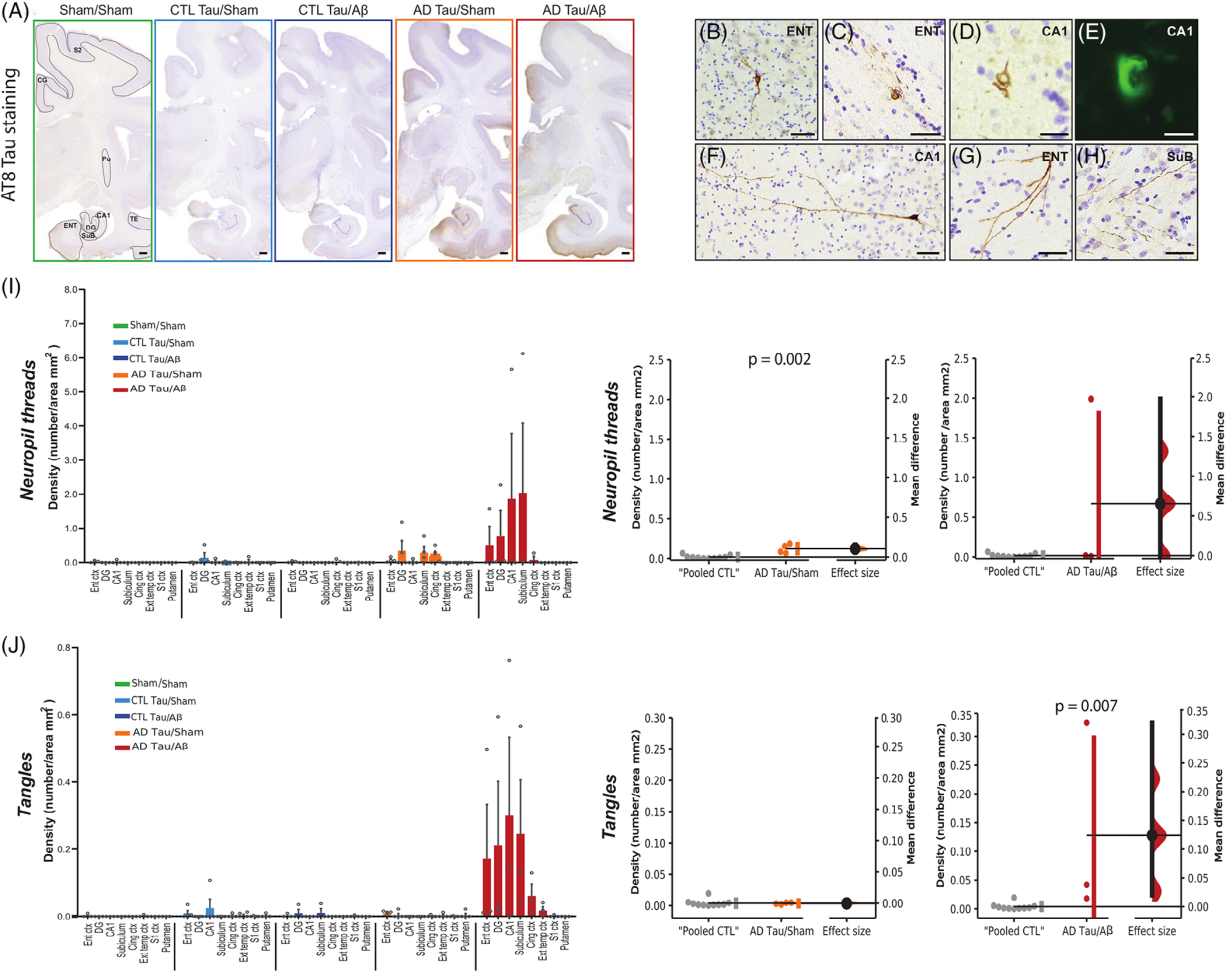
Injections of Alzheimer's disease (AD) patients‐derived tau aggregates induced tau pathology in macaques. (A) Representative images of AT8 staining in whole brain slides. Left slide: Segmentation of each region of interest (ROI) for quantitative analyses. CG: cingulate cortex, S2: somatosensory cortex, Pu: putamen, DG: dentate gyrus, SuB: subiculum, Ent: entorhinal cortex, and TE: temporal cortex. Scale bar: 1.45 mm. (B–H) Illustrative images of AT8‐positive lesions: (B–D) AT8‐positive neurofibrillary tangles, (E) mature tangle stained by thioflavin‐S, and (F–H) AT8‐positive neuropils threads. (I) Quantification of AT8‐positive neuropils density in different ROIs. (J) Quantification of AT8‐positive tangles density. Quantitative results are represented in the five experimental groups using bar and dot plots. They are also represented with estimation plots where sham animals and the two control (CTL)‐tau groups were pooled (“pooled CTL”) and compared to AD‐tau/sham or AD‐tau/Aβ groups. The *p*‐values refer to the results of Mann‐Whitney tests. Scale bar: 20 μm

Eighteen months after tau intracerebral injections, we euthanized the macaques after performing a lumbar puncture for CSF collection. To assess the seeding and spreading of tau pathology, we performed neuropathological examination using AT8‐staining at the injection site (entorhinal cortex), in connected brain areas (the dentate gyrus, CA1, the subiculum in the hippocampal trisynaptic loop; and the cingulate cortex, a projection site of the hippocampal neurons) and non‐connected brain areas (somatosensory cortex, putamen, external temporal cortex). The macroscopic assessment of whole‐brain hemisphere histological slides clearly showed positive AT8‐staining in the entorhinal cortex, the hippocampus, and the cingulate cortex of macaques injected with AD‐tau, with or without oligomeric Aβ injections (compared to CTL‐tau animals or sham) (Figure 3A). We found no AT8‐staining in brain regions not connected anatomically to the injection site in these animals. At the microscopic level, we found typical neuropil threads and neurofibrillary tangles (Figure 3B–H), including mature thioflavin‐S positive tangles (Figure 3E).

Quantitatively, we confirmed the occurrence of neuropil threads in macaques injected with AD‐tau (Figure 3I; Mann‐Whitney test: “pooled‐CTL” vs. AD‐tau/sham, *p* = 0.002). Interestingly, neurofibrillary tangles and mature tangles were found only in AD‐tau/Aβ macaques (Figure 3J; Mann‐Whitney test: “pooled‐CTL” vs. AD‐tau/Aβ, *p* = 0.007).

As expected, the density of neurofibrillary tangles was positively correlated with the concentration of p‐tau181 in the CSF (Spearman *r* = 0.42; *p* = 0.046).

### 3.5 Amyloid pathology

**FIGURE 4:**
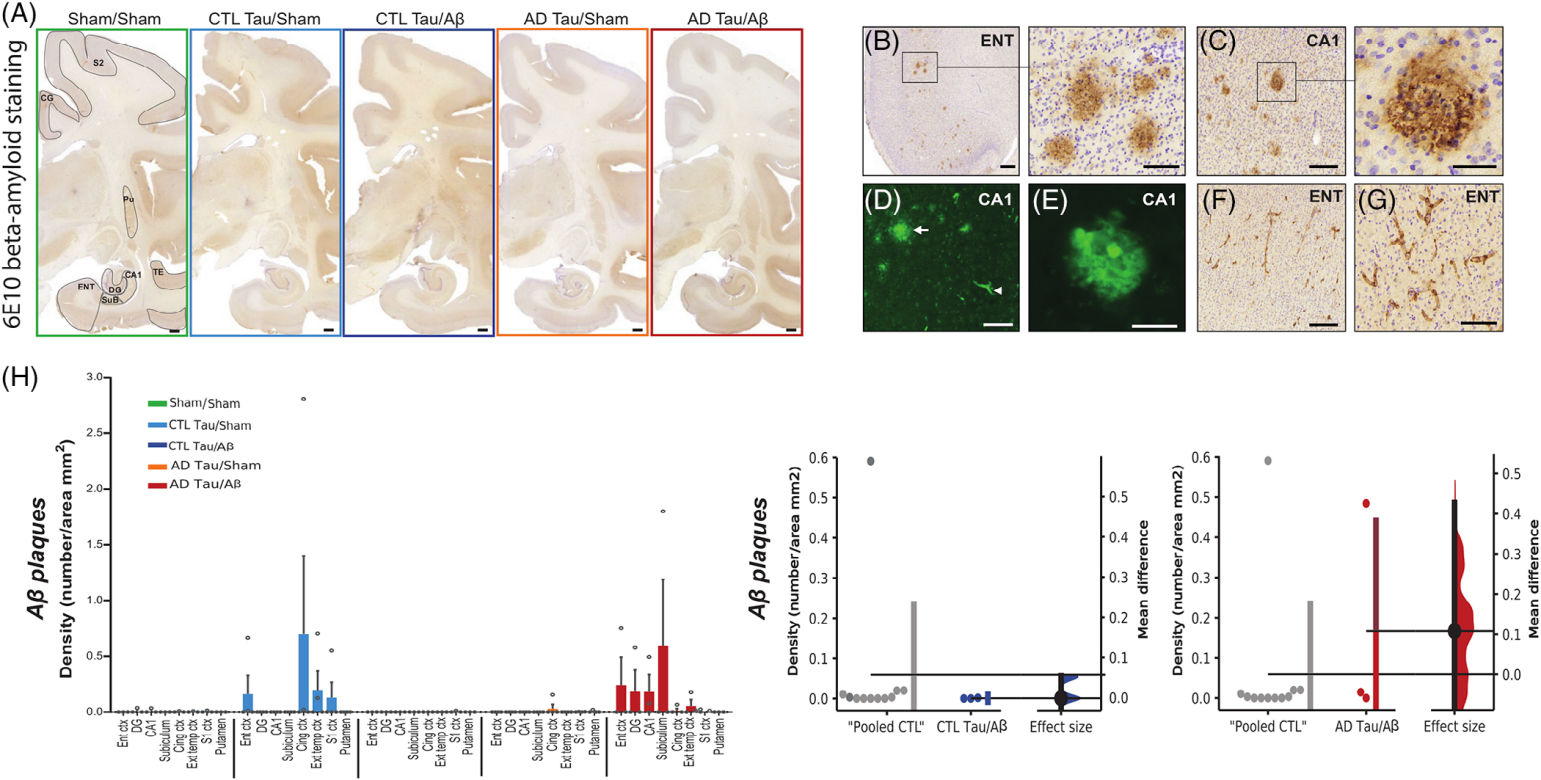
Injections of oligomeric amyloid‐β (Aβ) did not induce plaque formation but amyloid angiopathy. (A) Representative images of amyloid pathology in whole brain slides using 6E10 staining. Scale bar: 1.45 mm. (B, C) Amyloid plaques (6E10) were found in macaques with or without oligomeric Aβ injections. (D and E) Thioflavin‐S positive amyloid plaque (white arrow) and capillary (white arrowhead). (F, G) Cerebral amyloid angiopathy (6E10) was found only in macaques injected with oligomeric Aβ. (H) Quantitative analyses of amyloid plaque density. Quantitative results are represented in the five experimental groups using bar and dot plots. They are also represented with estimation plots where sham animals and the two CTL‐tau groups were pooled (“pooled CTL”) and compared to AD‐tau/sham or AD‐tau/Aβ groups. Scale bar: 20 μm

To determine the extent of Aβ pathology, we performed Aβ immunohistochemistry with 6E10 antibody (Figure 4A), which revealed both amyloid plaques (Figure 4B,C, including mature thioflavin S‐positive plaques, Figure 4D,E) and cerebral amyloid angiopathy (Figure 4F,G). While cerebral amyloid angiopathy was exclusively found in macaques injected with oligomeric Aβ, quantitative analyses of amyloid plaques did not show any difference between groups (Figure 4H). Indeed, “spontaneous” amyloid plaques were also found in CTL‐tau/sham animals, as previously described in mature macaques.^28^, ^49^

### 3.6 Glial cell reactivity

**FIGURE 5:**
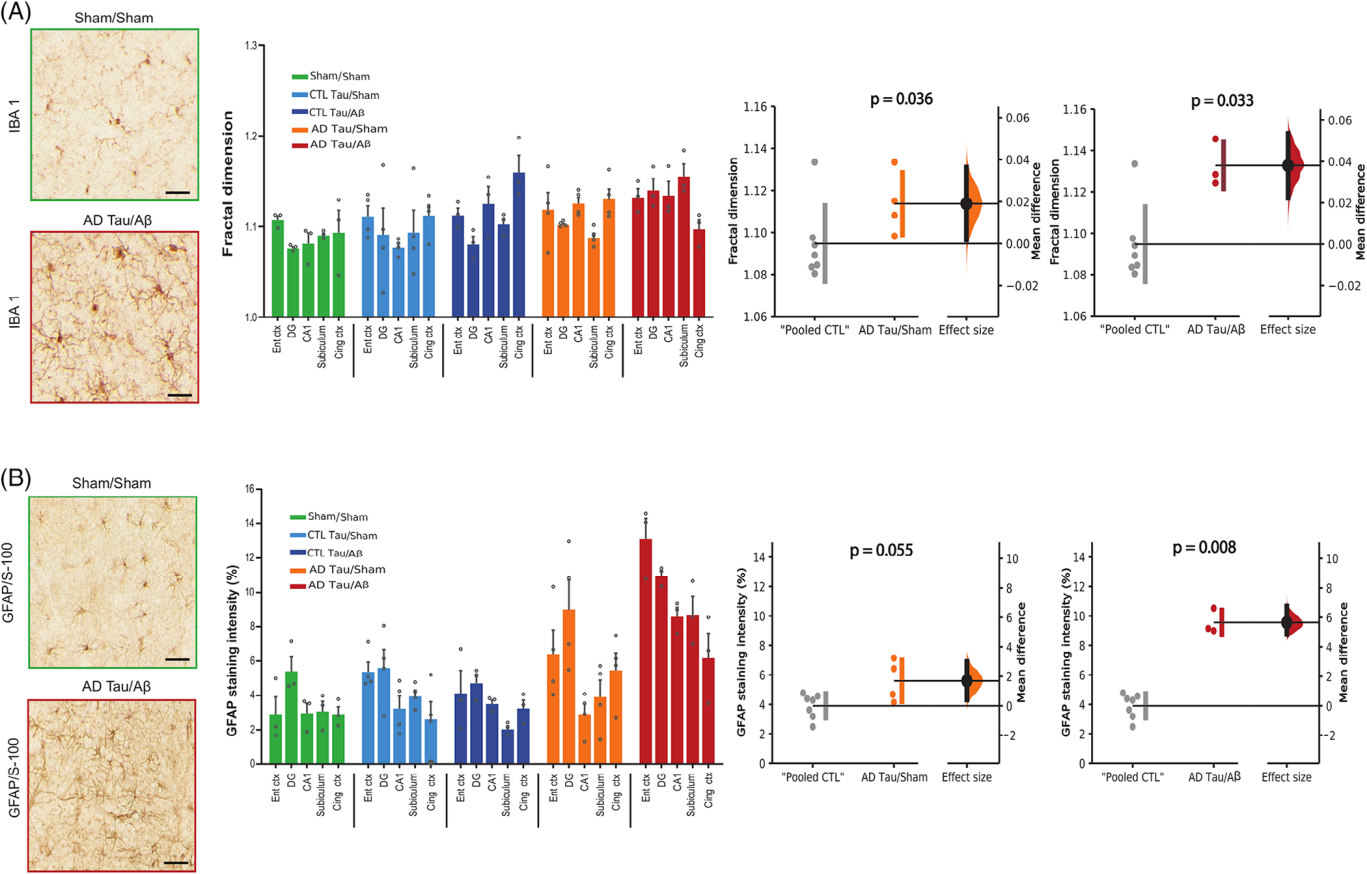
Injections of Alzheimer's disease (AD) patients‐derived tau aggregates induced brain inflammation. (A) Representative images of microglial staining (Iba‐1) and quantification of microglial fractal dimension for each experimental condition in brain areas affected by tauopathy. Right: Estimation plots of the quantification in the pooled region of interest. (B) Representative images of astrocytic staining (GFAP/S‐100) and quantitative analyses of the surface intensity of staining in brain areas affected by tauopathy. (A, B) Quantitative results are represented in each of the five experimental groups in brain areas affected by tauopathy using bar and dot plots. They are also represented with estimation plots showing quantification in all regions of interest. Sham animals and the two control (CTL)‐tau groups were pooled (“pooled CTL”) and compared to AD‐tau/sham or AD‐tau/Aβ groups. The *p*‐values refer to the results of Mann–Whitney tests. Scale bar: 20 μm

We then investigated whether brain areas affected by tauopathy (entorhinal cortex, dentate gyrus, CA1, subiculum, and the cingulate cortex) exhibit astrocytic and microglial reactivity. The fractal dimension of microglial cells (i.e., the complexity of ramifications^37^) was significantly increased in macaques injected with AD‐tau, with a stronger effect size in animals co‐injected with Aβ (Figure 5A; Mann‐Whitney test: “pooled CTL” vs. AD‐tau/sham, *p* = 0.036; “Pooled CTL” vs. AD‐tau/Aβ *p* = 0.033). Hyper‐ramification indicates a “pre‐activation” state of microglia cells that sense environmental changes.^50^, ^51^

Regarding astrocytes, we found higher GFAP/S‐100 surface staining intensities in both AD‐tau and AD‐tau/Aβ, with a more robust effect size in animals co‐injected with Aβ (Figure 5B; Mann‐Whitney test: “pooled CTL” vs. AD‐tau/sham, *p* = 0.055; “pooled CTL” vs. AD‐tau/Aβ *p* = 0.008).

Glial reactivity was positively correlated with tau pathology. Neurofibrillary tangles' density was correlated with microglial fractal dimension (Spearman *r* = 0.57; *p* = 0.0008) and GFAP/S‐100 surface staining intensity (Spearman *r* = 0.61; *p* = 0.005). The density of neuropil threads was correlated with GFAP/S‐100 staining (Spearman *r* = 0.64; *p* = 0.004).

### 3.7 Synaptic loss

**FIGURE 6:**
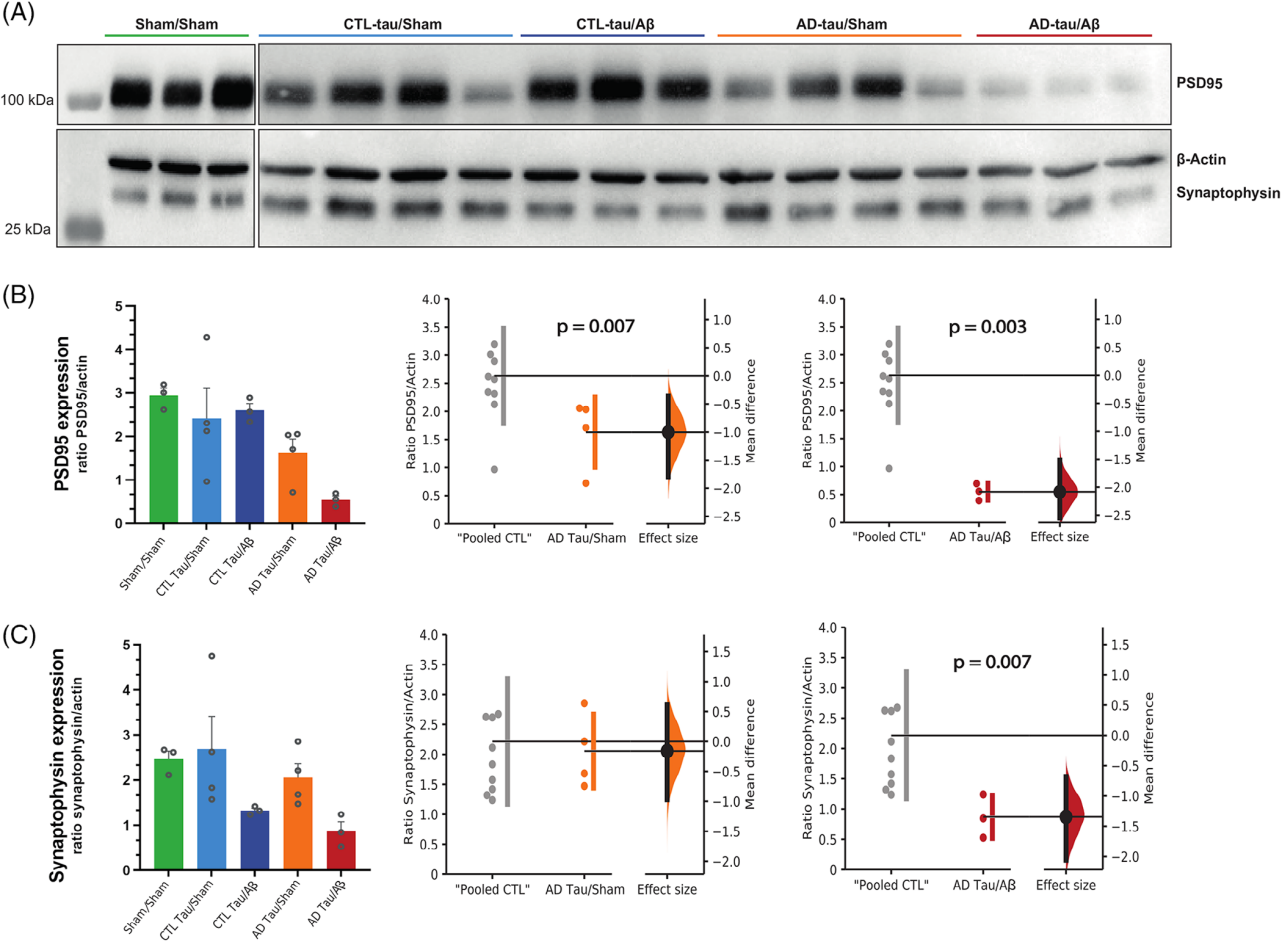
Injections of Alzheimer's disease (AD) patients‐derived tau aggregates induced synaptic loss in the CA1 subfield of the hippocampus. (A) Representative images of synaptophysin, β‐actin, and PSD95 immunoblotting for each macaque. (B, C) Quantitative analyses of PSD‐95 (B) and synaptophysin (C) expression level. Quantitative results are represented in the five experimental groups using bar and dot plots. They are also represented with estimation plots where sham animals and the two control (CTL)‐tau groups were pooled (“pooled CTL”) and compared to AD‐tau/sham or AD‐tau/Aβ groups. The *p*‐values refer to the results of Mann–Whitney tests

To determine whether brain injections of AD‐tau seeds in the entorhinal induced remote synaptic loss in CA1, a hippocampal subfield particularly affected by neurodegeneration in AD,^52^ we assessed with immunoblotting the expression of two presynaptic and postsynaptic proteins: synaptophysin and PSD95. PSD‐95 expression level was significantly decreased in macaques injected with AD‐tau, with a stronger effect size in animals co‐injected with Aβ (Figure 6A,B; Mann‐Whitney test: “pooled CTL” vs. AD‐tau/sham, *p* = 0.007; “pooled CTL” vs. AD‐tau/Aβ, *p* = 0.003). Synaptophysin expression level was significantly decreased in AD‐tau/Aβ macaques (Figure 6A,C; Mann‐Whitney test: “pooled CTL” vs. AD‐tau/Aβ, *p* = 0.007) but not in AD‐tau/sham macaques.

Interestingly, synaptic loss in CA1 was associated with neurofibrillary tangles density (Spearman *r* = −0.58; *p* = 0.008 for synaptophysin expression level and AT8 staining; and Spearman *r* = −0.79; *p* = 0.0001 for PSD‐95 expression level and AT8 staining). We found no significant association between synaptic loss and amyloid plaques or microglia reactivity. However, there was a significant negative correlation between astrocytic reactivity (GFAP/S100 surface staining) and PSD‐95 expression level (Spearman *r* = −0.78; *p* = 0.0002).

### 3.8 Neuronal loss

We then investigated whether brain injections of AD‐tau seeds induced neuronal loss in the stratum pyramidal and/or the stratum radiatum lacunosum molecular (SRLM) of CA1.^52^ Stereological neuronal counting of NeuN‐positive cells showed a non‐significant trend for neuronal loss in the two CA1 layers in AD‐tau/Aβ macaques only (Figure S1; stratum pyramidal: Mann‐Whitney test: “Pooled CTL” vs. AD‐tau/sham, *p* = 0.37; “Pooled CTL” vs. AD‐tau/Aβ, *p* = 0.19; SRLM: Mann‐Whitney test: “Pooled CTL” vs. AD‐tau/sham, *p* = 0.31; “Pooled CTL” vs. AD‐tau/Aβ, *p* = 0.19).

### 3.9 Proteomic analyses

**FIGURE 7:**
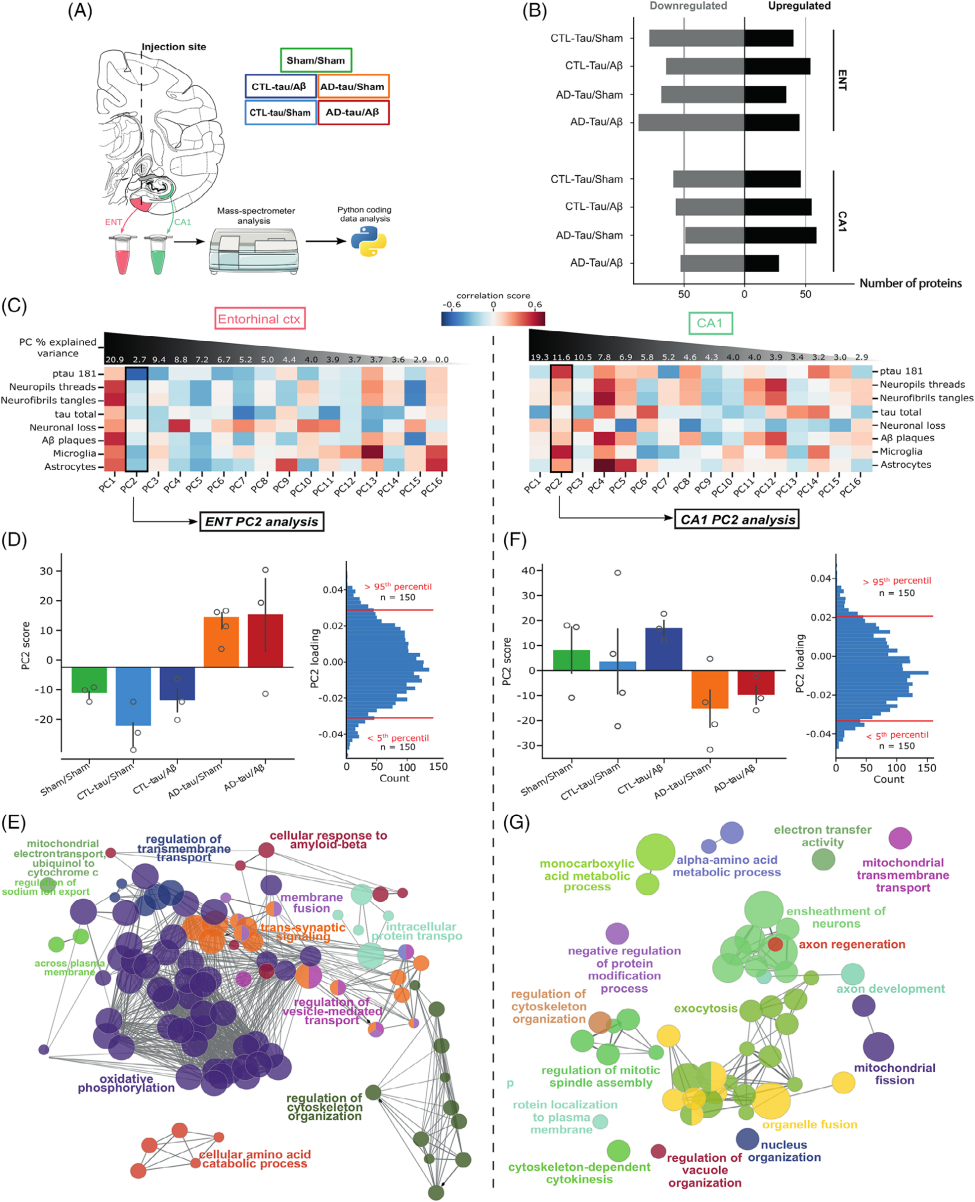
Proteomic analyses highlighted neuronal dysfunction and spreading‐related pathways in macaques. (A) Schematic of the overall design of proteomic analyses with experimental groups and regions (ENT: entorhinal cortex). (B) Summary of the differentially expressed proteins observed in the different groups and brain regions (reference: Sham/sham animals). (C) Heatmap of the correlation coefficient between pathological variables and principal component analysis computed on the whole proteome. Principal components (PCs) are ranked according to the amount of explained variance. The black square illustrates the PC selected for pathway enrichment analysis. (D, F) Individual scores on the selected PC per group (left). Distribution of the variable (i.e., proteins) loadings in the selected PC (right), red lines illustrate the boundaries for selecting relevant proteins. (E, G) Gene ontology network enrichment analysis. Node size illustrates the number of proteins belonging to a given term. Edge length illustrates the similarity between nodes. An edge‐weighted force‐directed layout was applied to cluster nodes by similarity

To further study the spatial response (local and distant) to tau seeds injections and its modulation by oligomeric Aβ, we performed mass spectrometry‐based proteomics of the macaques’ entorhinal cortex and CA1 (Figure 7A). Using pairwise comparisons, we found many proteins differentially regulated in the different experimental conditions (Figure 7B).

We used unsupervised dimensionality reduction by principal component analysis (PCA) to capture cross‐group proteome‐wide variations. This method provides a set of dimensions or axes (e.g., principal components) that capture coordinated variations in protein abundance and are ordered by decreasing the explained variance. We correlated these “proteome‐axes” with the neuropathological assessments described above. Interestingly, in both regions investigated, the first principal dimension only poorly correlated with neuropathological features, thus capturing variations in protein abundance not relevant to the observed phenomenon. On the contrary, we observed that PC2 (explaining 10.7% and 11.6% of proteome variations, respectively) strongly correlated with tau pathology, inflammation, and CSF‐pTau181 (Figure 7C) and discriminated between control and experimental groups (Figure 7D‐G). Such correlation profile suggests that the variations in protein abundance captured by PC2 likely contributed to or at least reflected the neuropathological process. To characterize the proteins contributing to this proteome‐axis, we selected the 10% proteins (under 5th and above 95th percentiles) contributing the most to these PCs for functional analysis.

Network analyses^40^ of PC2 top contributors showed that proteins involved in transport and phosphorylation regulation were the most differentially expressed in the entorhinal cortex of macaques injected with AD‐tau seeds, while proteins involved in exocytosis were the most dysregulated in CA1. It is consistent with seeding events around the injection site (entorhinal cortex) and distant spreading phenomenon involving tau exocytosis in CA1^13^, ^15^ (Figure 7D‐G). Disease‐related changes were relatively similar in AD‐tau/sham and AD‐tau/Aβ groups, confirming neuropathological findings suggesting that oligomeric Aβ injections had a limited impact on early seeding, spreading, and inflammatory events. Many synaptic proteins were identified in PC2 (36/300 = 12%), such as neurexins (NRXN2, NRXN3) and synaptotagmin (SYT7) for example. It reinforces our immunoblot results regarding synaptic impairment.

## 4 DISCUSSION

In this study, we extracted, purified, selected, and characterized tau aggregates from *post mortem* AD brains and equivalent fractions from aged‐matched healthy control brains. The injection of nanograms of these AD‐tau extracts in macaques’ brains, which possessed in vitro seeding activity compared to control extracts, increased the concentration of soluble p‐tau181 and induced the formation of neuropil threads. The same extract from control brains was biologically inert. In AD‐tau/sham and AD‐tau/Aβ macaques, tau pathology spread from the entorhinal to the hippocampal trisynaptic loop and the cingulate cortex, resuming the experimental progression of Braak stage I to stage IV in 18 months (as in humans, the macaque anterior cingulate has direct connections with the entorhinal cortex and the hippocampus^53^). Supporting the “prion‐like” hypothesis, tau pathology was not found in brain areas not anatomically connected to the entorhinal cortex.

Our findings are the first evidence of patient‐derived AD‐tau self‐propagation and dissemination in rhesus monkeys (*Macaca mulatta*), confirming previous works on transgenic and wild‐type rodents.^8^, ^9^, ^10^, ^11^ Further, it reproduces the pathophysiology of sporadic AD tauopathy more closely thanks to molecular and anatomical homology between humans and macaques. The present work also aligns with our previous study showing that tau seeds from patients with progressive supranuclear palsy (PSP, a primary 4R tauopathy) can trigger the pathological conversion of endogenous tau in macaques and the spreading of the pathology in connected brain areas.^43^ Interestingly, while the injection of PSP‐tau was sufficient to induce mature neuronal and glial lesions in our previous work, we found in this study tangles (AT8‐positive), mature tangles (thioflavin‐S positive), pre‐ and post‐synaptic loss (synaptophysin and PSD‐95 expression level), and neuronal death (increased CSF total‐tau concentration) only in AD‐tau macaques co‐injected with oligomeric Aβ. Thus, while AD tauopathy can replicate and spread autonomously in macaques, it requires oligomeric Aβ for maturation and neuronal toxicity, emphasizing the distinction between primary and secondary tauopathies. The effect of amyloid pathology on increasing tau‐induced neuronal damage has been reported in transgenic mice overexpressing mutant tau in the entorhinal cortex and crossed with APP/PS1 mice.^54^ However, an acceleration of tau pathology spread has also been observed in this transgenic mouse model, which we did not find in primates.

The injection of oligomeric Aβ in CTL‐tau macaques did not induce any tau pathology but amyloid angiopathy. There was no difference between groups regarding quantifying amyloid plaques, which could occur spontaneously in sham animals, as previously described in mature macaques.^49^ It suggests that oligomeric Aβ is insufficient to induce AD tauopathy, contrary to what might be expected in the context of the most “vertical” and “deterministic” interpretation of the amyloid cascade hypothesis.^55^ Indeed, an incipient tauopathy was a prerequisite for the pathological action of oligomeric Aβ. These findings support alternative models of sporadic AD, where amyloid and tau pathologies develop autonomously before their interaction.^4^ Our study cannot conclude regarding the mechanisms by which oligomeric‐Aβ promotes the maturation of ongoing tauopathy before the formation of amyloid plaques. Nevertheless, we can hypothesize that inflammation induced by oligomeric‐Aβ may enhance tau hyperphosphorylation by disrupting calcium regulation for instance.^56^ This hypothesis finds support in our observation of heightened glial reactivity in animals co‐injected with oligomeric‐Aβ, as well as the correlation we identified between glial reactivity and AT8 pathology. It would be interesting in the future to investigate in our experimental model whether other sources of inflammation in the central nervous system might have the same nonspecific effect on the maturation of tauopathy.

Beyond answering pathophysiological questions, our study makes it possible to establish what seems to be an animal model closest to early sporadic AD. First, our macaques exhibit typical AD lesions, including evidence of fibrillated (thioflavin‐S positive) tau pathology. Second, we found both astrocytic and microglial reactivity correlating with tau pathology, as in human pathology.^57^ Third, as expected in early AD, the concentration of CSF p‐tau181 (a well‐established biomarker for early AD‐tauopathy used in clinical practice^48^) correlated with tau tangles. Moreover, CSF total‐tau concentration (a standard marker of neurodegeneration^5^) increased only in the group of macaques exhibiting mature neurofibrillary tangles (frequently associated with local neurodegeneration in AD). Fourth, as is often reported in the early stages of AD, we found pre‐ and postsynaptic degeneration in CA1 without overt neuronal loss. Interestingly, this synaptic impairment was mediated in our study by tau rather than Aβ and appears to involve astrocytes rather than microglia, as has recently been reported in mouse and human pathology.^58^, ^59^ Fifth, the proteomic analyses in the entorhinal cortex of macaques (the injection site) and CA1 (a remote projection area) were consistent with published proteomic studies in AD cases.^60^ For instance, among principal components highly correlated with neuropathological findings, we found dysregulations of proteins involved in oxidative phosphorylation, membrane transport, cytoskeleton organization, catabolic processes, and amyloid pathway, as in cortical samples of patients with very early (asymptomatic and prodromal) AD.^61^, ^62^ We also found alterations of proteins involved in mitochondrial functions in macaques, as reported in later stages of the disease (patients with AD dementia).^61^ Disease‐related changes were relatively similar in AD‐tau/sham and AD‐tau/Aβ groups, confirming neuropathological findings suggesting that oligomeric Aβ injections had a few impacts on early tau pathology development.

Interestingly, proteins involved in phosphorylation and transport regulation were the most altered around the injection site of AD‐tau seeds (the entorhinal cortex), while proteins involved in exocytosis were the most dysregulated in a remote projection site (CA1), supporting “prion‐like” mechanisms. Taken together, AD‐tau injected macaques are promising animal models for an efficient transition from preclinical research in transgenic mice to the first phases of clinical trials in humans.^28^ An alternative approach to model early AD in macaques consists of the viral delivery of human‐mutated tau in the entorhinal cortex. This strategy can induce the templating and propagation of tau pathology, inflammation, and alteration in CSF biomarkers.^63^ However, it requires the injection of adeno‐associated virus (AAV) expressing two tau mutations causing familial frontotemporal dementia, which appears interesting but is arguably less relevant to study sporadic AD than our model based on brain extracts from sporadic AD patients. In the years to come, we hope that these two macaque models, with their respective strengths and weaknesses, will prove to be complementary for the development of new treatments for primary and secondary tauopathies.

Beyond these strengths, our study has some limitations. Although we observed mature neurofibrillary tangles in macaques, we must acknowledge that lesions were sparse compared to what is seen in human pathology. Moreover, few AT8‐positive lesions were found to be thioflavin‐S positive, suggesting that we have mainly quantified earlier tau lesions, including pretangles and soluble forms of phosphorylated‐tau. This does not weaken our results, as soluble phosphorylated‐tau is likely responsible for tau spreading between neurons in aged macaques and humans and may be the forerunner to tangles.^56^, ^64^ Future analyses using for instance immunostaining with MC‐1 and PHF‐1 antibodies could allow us to better characterize the maturity levels of tangles in our model.^65^ It is also important to note that the macaque brains were not perfusion‐fixed but post‐fixed in our study, allowing us to preserve one fresh‐frozen hemisphere for proteomic analyses. This approach contrasts what is most commonly done in animal studies, including previous macaque studies. It may have reduced the amount of early‐stage tau pathology still visible, as tau undergoes rapid dephosphorylation *post mortem*. We also acknowledge that neuronal death associated with tauopathy is not striking in our model. Indeed, although we described synaptic degeneration and an increase in CSF total‐tau concentration in AD‐tau/Aβ macaques, stereological counting of pyramidal neurons in CA1 showed only a non‐significant trend towards neuronal loss in this hippocampal subfield particularly vulnerable to AD pathology.^52^ However, counting NeuN‐labeled nuclei is an insensitive measure associated with late‐stage pathology, as degeneration begins in the dendrites, not the soma, in both humans with AD^66^ and aged macaques.^64^ Altogether, it suggests that our primate model reflects the early stages of AD and can arguably be optimized to model later stages by using, for instance, more concentrated and seeding‐competent brain extracts thanks to other protocols for tau seeds extraction and purification.^10^ One can also imagine letting the macaques age longer after tau seeds injections. This study also lacks behavioral experiments. One of the significant advantages of working with macaques is using memory tests very similar to those used in humans^36^ to assess AD‐related cognitive impairment. However, it requires months of habituation for each animal, which makes it very difficult to achieve in such a large macaque study (*n* = 17, a significant strength of the study). It will be a challenge for future studies using this model. Finally, another limitation of this study is the small sample size within each experimental group (*n* = 3 or 4), despite our large cohort (*n* = 17). This choice allowed us to explore as many experimental combinations as possible but we could not analyze them with appropriate statistical regression analyses, due to the lack of statistical power. However, it was our a priori decision to study the impact of AD‐tau and its interaction with Aβ, which is why we grouped the control animals in our statistical analyses.

In conclusion, our novel experimental approach in non‐human primates allowed us to experimentally support Braak's tauopathy progression model to confirm the “prion‐like” hypothesis of AD tauopathy in an anthropomorphic brain and to establish a new animal model of sporadic AD close to the human pathology. Contrary to some expectations, we observed a role for oligomeric Aβ in the maturation of tau pathology rather than in its ability to replicate and spread. Together, these results pave the way for new therapeutic innovations in AD.

## AUTHOR CONTRIBUTIONS

Chuan Qin, Erwan Bezard, and Vincent Planche designed the experiments and supervised the overall study. Benjamin Dehay and Vincent Planche extracted patient‐derived tau seeds. Taxiarchis Katsinelos and William A. McEwan performed in vitro seeding experiments. Erwan Bezard and Vincent Planche performed macaque surgeries. Morgane Darricau performed histological experiments. Remi Kinet, Aurélie Bedel, Stéphane Claverol, Caroline Tokarski, and Mathieu Bourdenx performed biochemical and proteomic analyses. Changsong Dou, Tao Zhu, Li Zhou, Xianglei Li, Ling Zhang, Ran Gao, and Chuan Qin supervised macaque experiments and collected fluids and tissues. Morgane Darricau and Vincent Planche wrote the first draft of the manuscript. All authors discussed the results and participated in manuscript editing.

## CONFLICT OF INTEREST STATEMENT

Erwan Bezard is a director and shareholder of Motac Neuroscience Ltd. During the past 3 years, Vincent Planche was a local unpaid investigator or sub‐investigator for clinical trials granted by Novo Nordisk, Biogen, TauRx Pharmaceuticals, Janssen, Green Valley Pharmaceuticals, and Alector. Vincent Planche served as a consultant for Motac Neuroscience Ltd, outside the submitted work. The other authors declare no conflict of interest. Author disclosures are available in the supporting information.

## CONSENT STATEMENT

Human brain samples were obtained from the Netherlands Brain Bank (Department of Pathology, Amsterdam UMC) and the French Brain Bank GIE NeuroCEB (Pitié‐Salpétrière Hospital, Paris). According to Dutch and French ethical guidelines, consent was signed by the patients or their next of kind in their name.

## Supporting information

Supporting information

Supporting information
