# Supplementary material for: Tau seeds from Alzheimer's disease brains trigger tau spread in macaques while oligomeric‐Aβ mediates pathology maturation
Source: Alzheimers Dement. 2023 Dec 26;20(3):1894–912. doi: 10.1002/alz.13604 (PMC10984505; doi:10.1002/alz.13604)
Supplement: Supplementary file 2 — Supporting information [file ALZ-20-1894-s001.pdf]

Supplementary Materials for :

Tau seeds from Alzheimer’s disease brains trigger tau spread in macaques while oligomeric-Aβ mediates pathology maturation

Darricau, Dou, et al.

It includes:

- Supplementary table 1
- Supplementary table 2
- Supplementary figure 1

Supplementary Table 1. Brain samples used in this study.

Samples	Disease condition	Age (years)	Sex	Post-mortem interval (hours)	Braak stage	Regions	Origin
A	AD	90	F	3:50	5	Sup. Frontal gyrus	Netherlands Brain Bank
B	AD	87	F	6:20	5	Parahippocampal gyrus	
F	AD	90	F	3:10	5	Sup. Frontal gyrus	
G	AD	89	F	2:55	5	Hippocampus	
H	AD	84	F	4:00	6	Amygdala	
J	AD	86	F	3:20	5	Hippocampus	
L	AD	97	F	3:15	6	Amygdala	
M	AD	87	F	6:55	5	Parahippocampal gyrus	
N	AD	87	F	6:55	5	Sup. Frontal gyrus	
R	AD	82	F	4:15	5	Amygdala	
S	AD	87	F	6:20	5	Sup. Frontal gyrus	
T	AD	92	F	4:45	5	Amygdala	
E	CTL (non-demented)	88	M	5:40	4	Sup. Frontal gyrus	
I	CTL (non-demented)	87	M	10:20	1	Sup. Frontal gyrus	
K	CTL (non-demented)	86	F	6:25	2	Sup. Frontal gyrus	
O	CTL (non-demented)	86	F	/	/	Amygdala	Paris NeuroCEB
P	CTL (non-demented)	86	F	/	/	Cingulate cortex	
Q	CTL (non-demented)	84	H	/	/	Amygdala	

Supplementary Table 2. Individual description of the macaques included in this study

Macaque #	Sex	Weight (kg)	Age (years)	Groups
1	M	7.6	13	Sham/Sham
2	M	5.2	15	Sham/Sham
3	F	5.5	16	Sham/Sham
4	F	5.6	16	CTL-tau/Sham
5	F	5.7	15	CTL-tau/Sham
6	M	8.5	15	CTL-tau/Sham
7	M	7.9	14	CTL-tau/Sham
8	F	4.5	16	CTL-tau/Aß
9	F	6.3	14	CTL-tau/Aß
10	M	8.1	14	CTL-tau/Aß
11	F	6.1	14	AD-tau/Sham
12	M	8.3	17	AD-tau/Sham
13	M	6.2	15	AD-tau/Sham
14	F	5.2	14	AD-tau/Sham
15	M	6.2	15	AD-tau/Aß
16	F	5.2	16	AD-tau/Aß
17	F	7.3	15	AD-tau/Aß

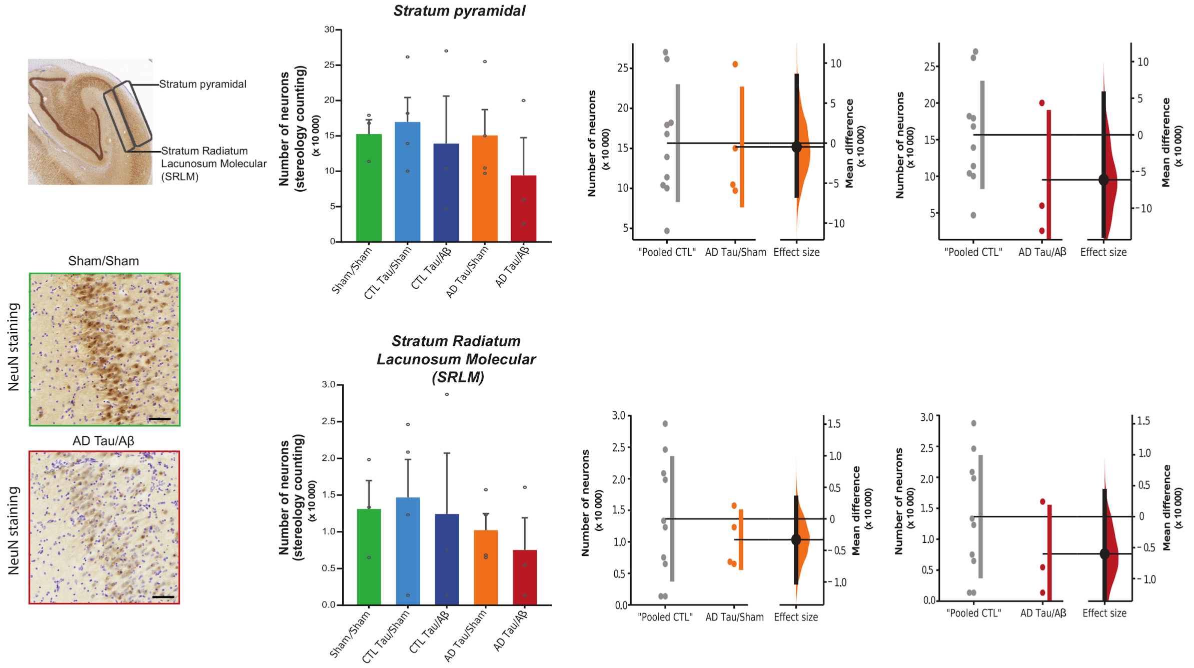

Suppl. Fig. 1. Injections of AD patients-derived tau aggregates induced a trend for neuronal loss in the CA1 subfield of the hippocampus. Illustrative images of NeuN immunohistochemistry in CA1 and stereological quantification of NeuN-positive neurons in the stratum pyramidal and stratum radiatum lacunosum molecular (SRLM) of CA1. Quantitative results are represented in the 5 experimental groups using bar and dot plots. They are also represented with estimation plots where sham animals and the 2 CTL-tau groups were pooled ("pooled CTL") and compared to AD-tau/Sham or AD-tau/Aβ groups. The *p-values* refer to the results of Mann-Whitney tests. Scale bar: 20 µm.
